# Spinal pain prevalence in childhood and socioeconomic patterns: a comparative study in four European birth cohorts

**DOI:** 10.1007/s00431-026-07461-w

**Published:** 2026-10-03

**Authors:** Line Marie Toft Dyhr, Anne-Marie Nybo Andersen, Berna Araújo, Tim Cadman, Luise Cederkvist, Eveliina Heikkala, Lise Hestbæk, Jaro Karppinen, Raquel Lucas, Iiro Nerg, Costanza Pizzi, Maja Popovic, Lorenzo Richiardi, Justiina Ronkainen, Sylvain Sebert, Makram Talih, Demetris Avraam

**Affiliations:** 1 Department of Public Health, University of Copenhagen, Copenhagen, Denmark; 2 University of Porto, Porto, Portugal; 3 University Medical Centre Groningen, Groningen, the Netherlands; 4 Barcelona Institute for Global Health, Barcelona, Spain; 5 Research Unit of Health Sciences and Technology, University of Oulu, Oulu, Finland; 6 Wellbeing Services County of Lapland, Rovaniemi, Finland; 7 Department of Sports Science and Clinical Biomechanics, University of Southern Denmark, Odense, Denmark; 8 Research Unit of Population Health, University of Oulu, Oulu, Finland; 9 Department of Medical Sciences, University of Turin, Turin, Italy

**Keywords:** Spinal pain, Socioeconomic inequalities, Birth cohorts, Children, Adolescents

## Abstract

The aim of this study was to examine the prevalence and socioeconomic patterns of spinal pain in children and adolescents across different European contexts. We used individual participant data from four population-based birth cohorts from Denmark, Portugal, Italy, and Finland. Data on anatomical location, frequency, intensity, and pain-related functional limitations of spinal pain in children aged 7–18 years were analysed. Maternal educational level at birth was used as an indicator of the child’s socioeconomic background. Socioeconomic patterns in spinal pain were quantified by the slope and relative indices of inequality. The prevalence of spinal pain varied from 4.6% (previous 3 months) in Portuguese 7-year-old boys to 92.3% (previous 12 months) in Danish 18-year-old girls. Prevalence increased with age, and females reported more pain than males. Among children reporting spinal pain, approximately 59% experienced frequent pain, 59% intense pain, and 35% pain-related functional limitations, with notable differences across cohorts and age groups. We found no consistent socioeconomic gradient in the prevalence of spinal pain. However, among children reporting spinal pain, socioeconomic differences were more apparent in pain frequency, intensity, and daily-life functional limitations, although the direction and magnitude of these gradients varied across cohorts. *Conclusion*: Spinal pain is common in children and adolescents, but no consistent socioeconomic pattern was observed in its occurrence. Spinal pain appears prevalent across all socioeconomic groups, with inequalities emerging more clearly in the frequency, severity, and functional impact of pain, although these patterns varied across settings.

**Table:** 

**What is Known:** • *Paediatric spinal pain is common, but prevalence estimates vary widely across studies.* • *Evidence on socioeconomic inequalities in childhood spinal pain is mixed and inconsistent.*
**What is New:** • *Prevalence varies markedly across cohorts reflecting both methodological and contextual differences.* • *Inequalities in pain frequency, intensity, and daily-life impact are more apparent than those in spinal pain prevalence.*

**Supplementary Information:**

The online version contains supplementary material available at https://doi.org/10.1007/s00431-026-07461-w.

## Introduction

Spinal pain, defined as pain in the neck, mid-back, or low-back, is a major public health concern associated with substantial personal, societal, and economic consequences [1–4]. Although it was long viewed as a condition mainly affecting adults, growing evidence indicates that spinal pain often begins in childhood [5–7]. Spinal pain is common among children and adolescents [5, 8, 9], although prevalence estimates vary widely across studies, likely due to differences in measurement instruments, study periods, and population characteristics. A systematic review and meta-analysis reported a back pain prevalence of 19.1% (95% CI 16.6–21.7%) among children and adolescents [10], while other studies have reported estimates ranging from 3% to more than 50%, depending on age, pain definition, recall period, and population [11–15]. Evidence also indicates that spinal pain typically emerges around the age of 10–12 years and increases in prevalence with age during adolescence, approaching adult prevalence levels by late adolescence [5, 9, 16–18].

Spinal pain during childhood and adolescence can adversely affect daily functioning, including school attendance, participation in physical activities, sleep, and quality of life [7, 14, 19]. It is also associated with stress and poor general wellbeing [20]. Longitudinal studies indicate that spinal pain persists over time, with evidence that spinal pain in pre-adolescents almost doubles the risk of spinal pain in late adolescence [21], and that spinal pain in childhood is associated with an increased risk of spinal pain in adulthood [17, 21–23].

Given its early onset, persistence, and impact on daily life, it is also important to understand whether spinal pain is socially patterned. Research in adults indicates a consistent association between lower socioeconomic position and an increased risk of spinal pain [24]. However, research on socioeconomic inequalities in childhood spinal pain is more limited and has produced mixed findings [8, 12, 13, 25–27]. Some studies report higher prevalence among children of parents with lower educational attainment or income, whereas others find weak or no socioeconomic differences [25, 28–30]. These inconsistencies may partly reflect differences in socioeconomic indicators, pain definitions, and study populations.

Socioeconomic inequalities in child health may vary across countries because of political, institutional, and cultural contexts [31]. Cross-country comparisons may help determine whether observed socioeconomic gradients in spinal pain are consistent across settings or vary according to context. To our knowledge, no previous study has conducted a harmonised, cross-cohort analysis of socioeconomic inequalities in spinal pain during childhood and adolescence. We therefore applied a common harmonisation protocol across four European birth cohorts and used federated individual-level data to examine socioeconomic inequalities in spinal pain prevalence, frequency, intensity, and pain-related functional limitations.

## Methods

### Participating cohorts

We used data from birth cohorts participating in the EU Child Cohort Network, a federated infrastructure enabling harmonised cross-cohort analyses [32]. Cohorts were eligible if they had data on spinal pain collected between 6 and 19 years of age and information on maternal education at birth. Four cohorts met these criteria: the Danish National Birth Cohort (DNBC, Denmark) [33, 34], Geração 21 (G21, Portugal) [35], Nascita e INFanzia: gli Effetti dell’Ambiente (NINFEA, Italy) [36], and the Northern Finland Birth Cohort 1986 (NFBC1986, Finland) [37–40]. Additional descriptions are provided in Supplementary A.

### Outcome

Spinal pain was assessed using different instruments across cohorts. DNBC and NINFEA used the Young Spine Questionnaire [41], G21 used the Luebeck Pain Questionnaire [14], and NFBC1986 used spinal pain-related questions embedded in cohort questionnaires. DNBC contributed data from the 11-year (lifetime prevalence) and 18-year (12-month prevalence) follow-ups, G21 from the 7-, 10-, and 13-year follow-ups (3-month prevalence), NINFEA from the 13-year follow-up (lifetime prevalence), and NFBC1986 from the 16- and 18-year follow-ups (6-month prevalence).

Items from the questionnaires were harmonised into ten variables covering pain in three anatomical locations (neck, mid-back, and low-back), any spinal pain (pain in at least one location), high pain frequency, high pain intensity, three daily-life functional limitations (care-seeking behaviour, physical activity restriction, and school absenteeism), and any functional limitation (at least one of the three limitations). Pain frequency was harmonised into a binary variable indicating high frequency, with responses such as ‘once or twice’ classified as no and ‘often’ as yes. Pain intensity was similarly harmonised into a binary variable indicating high intensity, with ‘mild’ or ‘moderate’ pain classified as no and ‘severe’ pain as yes. Not all variables were available in all cohorts. Detailed questionnaire items and harmonisation procedures are provided in Supplementary B and C. The harmonised variables were analysed remotely on secure cohort servers using a federated analysis platform.

### Exposure

Maternal education at birth was used as the indicator of children’s socioeconomic background [31]. It was harmonised across cohorts using the International Standard Classification of Education 1997 (ISCED-97) into three levels: low (no education to lower secondary; ISCED-97 categories 0–2), medium (upper to post-secondary; ISCED-97 categories 3–4), and high (bachelor’s degree and above; ISCED-97 categories 5–6) [42]. Maternal education was converted into a scaled variable representing the midpoint of each category’s cumulative proportion within each cohort, accounting for differences in educational distributions and enabling cross-cohort comparisons [43]. Further details are provided in Supplementary D.

### Analysis

Descriptive statistics were generated for spinal pain prevalence, stratified by child’s sex and cohort at each follow-up. Cases with missing values were excluded from univariate and bivariate analyses (Supplementary E). Socioeconomic inequalities were assessed using the Slope Index of Inequality (SII) and the Relative Index of Inequality (RII) [43, 44]. The SII represents the absolute inequality in prevalence across the socioeconomic distribution, expressed as the difference in predicted prevalence between the highest and lowest socioeconomic ranks. The RII represents the corresponding relative inequality, expressed as the ratio of predicted prevalence at the highest and lowest socioeconomic ranks. Both measures were estimated using linear regression models with scaled maternal education as the socioeconomic exposure. An SII of 0 and RII of 1 indicate no inequality. These measures facilitate comparisons across populations with different socioeconomic distributions. Methods for calculating SII, RII, and their 95% confidence intervals (CI) are provided in Supplementary F.

Each cohort follow-up wave was analysed independently, with descriptive statistics and SII/RII estimates calculated separately for each cohort and follow-up. Repeated measurements from the same participants were not combined. All analyses were conducted using DataSHIELD, a privacy-preserving platform that enables federated analysis without transferring individual-level data to the analyst; only non-disclosive summary statistics were returned [45, 46].

## Results

### Prevalence of spinal pain

Marked variation in the prevalence of spinal pain was observed between cohorts and across follow-ups within cohorts (Table 1). In DNBC, the lifetime prevalence of any spinal pain at age 11 was 67.9%, while the 12-month period prevalence at age 18 was 87.9%. In G21, the 3-month period prevalence of any spinal pain ranged from 4.9% at age 7 to 16.1% at age 13. In NINFEA, 77.1% of children reported spinal pain by age 13. In NFBC1986, the 6-month period prevalence ranged from 59.9% at age 16 to 75.3% at age 18. The highest prevalence of any spinal pain was observed among 18-year-old females in DNBC (12-month period prevalence = 92.3%), and the lowest among 7-year-old males in G21 (3-month period prevalence = 4.6%).

**Table 1:** Prevalence (%) of spinal pain by sex and follow-up in the four participating cohorts

Follow-up		DNBC		G21			NINFEA ^c^	NFBC1986	
		11 y	18 y	7 y	10 y	13 y	13 y	16 y	18 y
Median age [range]		11 [11, 12]	18 [18–18]	7 [7, 8]	10 [10, 11]	13 [13, 14]	13 [13–13]	15 [15, 16]	17 [17, 18]
Lifetime or period prevalence		Lifetime	12-month period	3-month period	3-month period	3-month period	lifetime	6-month period	6-month period
Sample size ^a^	Boys	22,316	18,435	3003	2781	2359	756	3198	843
	Girls	24,362	26,245	2810	2571	2205	745	3506	1034
	Total	46,678	44,680	5813	5352	4564	1501	6704	1877
Any spinal pain (yes)	Boys	65.5	81.7	4.6	7.7	15.3	73.9	51.4	64.3
	Girls	70.2	92.3	5.3	8.6	16.9	80.3	67.7	84.2
	Total	67.9	87.9	4.9	8.1	16.1	77.1	59.9	75.3
Neck pain (yes)	Boys	56.2	66.8	0.4	0.6	1.0	61.5	34.1	49.0
	Girls	60.1	81.5	0.6	0.7	1.0	66.7	49.8	73.6
	Total	58.2	75.4	0.5	0.6	1.0	64.1	42.3	62.6
Mid-back pain (yes)	Boys	33.5	53.4	n/a	n/a	n/a	42.1	n/a	n/a
	Girls	37.7	64.9	n/a	n/a	n/a	46.1	n/a	n/a
	Total	35.6	60.1	n/a	n/a	n/a	44.1	n/a	n/a
Low-back pain (yes)	Boys	24.0	57.1	n/a	n/a	n/a	33.4	35.9	46.7
	Girls	32.0	78.3	n/a	n/a	n/a	51.8	49.3	62.2
	Total	28.2	69.5	n/a	n/a	n/a	42.6	42.9	55.2
Back pain (yes) ^b^	Boys	n/a	n/a	4.2	7.2	14.9	n/a	n/a	n/a
	Girls	n/a	n/a	4.7	8.0	16.3	n/a	n/a	n/a
	Total	n/a	n/a	4.4	7.6	15.6	n/a	n/a	n/a

*n/a*, not available

^a^ The sample size corresponds to the number of children with data on any spinal pain, while the prevalence of pain at a specific anatomical location is based on the non-missing data for each case

^b^G21 has a composite ‘back pain’ variable of either mid- or low-back pain

^c^The NINFEA 13-year follow-up is still ongoing, and the results presented are based on data from database version 2024.01 and therefore include only a subset of the cohort

Across cohorts with multiple follow-ups, spinal pain prevalence increased with age for all anatomical locations. Neck pain was the most common type of spinal pain across most age groups, followed by low-back pain at older ages. Sex differences were observed for nearly all anatomical locations, with higher prevalence among females. In DNBC, these differences appeared to widen between ages 11 and 18, although the different recall periods limit direct comparison. In G21, sex differences remained relatively stable across follow-ups, while in NFBC1986, spinal pain at all anatomical locations was more common among females at both ages 16 and 18.

### Spinal pain frequency, intensity, and functional limitations

Among children reporting any spinal pain, the prevalence of high pain frequency, high intensity, and pain-related functional limitations varied across cohorts and age groups (Table 2). In DNBC, 40.4% of children with spinal pain at age 11 and 76.0% at age 18 reported high pain frequency. The prevalence of high intensity was 60.8% and 71.2% at the two ages, respectively. In G21, the prevalence of high pain frequency was 48.3%, 48.8%, and 65.3% at ages 7, 10, and 13, respectively, while the prevalence of high intensity ranged from 28.1% at age 7 to 79.3% at age 13. However, these estimates should be interpreted with caution, because the proportion of children reporting any spinal pain was low in G21 (ranging between 3.3% and 8.5%) and only a subset had information on frequency, intensity, and functional limitations (Figure S1 and Table S1 in Supplementary E). In NINFEA, 66.7% reported high pain frequency and 52.7% high intensity. In NFBC1986, 69.8% and 69.5% of adolescents at age 18 reported high pain frequency and high intensity, respectively, while data at age 16 were not available. Girls generally had higher prevalences of high pain frequency and intensity than boys, except for high intensity in G21 at age 7. These sex differences were descriptive, as no formal statistical comparisons were performed.

**Table 2:** Prevalence of high pain frequency, high intensity, and functional limitations among children who reported any spinal pain, by sex and follow-up in the four participating cohorts

		DNBC		G21			NINFEA ^b^	NFBC1986	
Follow-up		11 y	18 y	7 y	10 y	13 y	13 y	16 y	18 y
Sample size ^a^	Boys	14,609	15,063	137	214	362	559	1643	542
	Girls	17,108	24,214	148	220	373	598	2375	871
	Total	31,717	39,277	285	434	735	1157	4018	1413
High frequency (yes)	Boys	37.6	65.3	35.7	47.5	65.3	61.7	n/a	57.7
	Girls	42.8	82.7	59.4	50.0	65.4	71.4	n/a	77.3
	Total	40.4	76.0	48.3	48.8	65.3	66.7	n/a	69.8
High intensity (yes)	Boys	58.5	59.4	54.5	46.7	74.5	49.6	n/a	60.3
	Girls	62.7	78.6	14.3	54.3	84.0	55.6	n/a	75.3
	Total	60.8	71.2	28.1	50.8	79.3	52.7	n/a	69.5
Any functional limitations (yes)	Boys	31.3	42.7	n/d	n/d	n/d	27.2	10.7	53.1
	Girls	32.8	53.7	n/d	n/d	n/d	29.6	11.7	54.2
	Total	32.1	49.5	n/d	n/d	n/d	28.4	11.3	53.8
Care seeking behaviour (yes)	Boys	13.6	21.1	n/d	n/d	n/d	13.4	10.7	26.0
	Girls	14.4	29.7	n/d	n/d	n/d	17.5	11.7	36.7
	Total	14.0	26.4	n/d	n/d	n/d	15.6	11.3	32.6
Physical activity restriction (yes)	Boys	21.3	33.2	n/d	n/d	n/d	19.1	n/a	48.1
	Girls	22.2	41.9	n/d	n/d	n/d	19.0	n/a	39.5
	Total	21.8	38.5	n/d	n/d	n/d	19.0	n/a	42.9
School absenteeism (yes)	Boys	9.6	9.2	n/d	n/d	n/d	4.3	n/a	n/a
	Girls	10.7	16.1	n/d	n/d	n/d	5.0	n/a	n/a
	Total	10.2	13.4	n/d	n/d	n/d	4.7	n/a	n/a

*n/a*, not available; *n/d*, not disclosed (i.e. for data protection any information related to small number of individuals is not disclosed)

^a^The sample size corresponds to the number of children with any spinal pain, while the prevalence of frequency, intensity or functional limitations is based on the non-missing data for each case

^b^The NINFEA 13-year follow-up is still ongoing, and the results presented are based on data from database version 2024.01 and therefore include only a subset of the cohort

The prevalence of pain-related functional limitations also varied across cohorts and age groups. In DNBC, 32.1% of children with spinal pain reported at least one functional limitation at age 11, with the proportion increasing to 49.5% at age 18. Physical activity restriction was the most common limitation at both ages. Prevalences of functional limitations were not estimated in G21 because of insufficient data. In NINFEA, 28.4% reported at least one functional limitation, with physical activity restriction again being the most common. In NFBC1986, 11.3% reported care seeking due to spinal pain at age 16, while 53.8% reported at least one limitation at age 18, with physical activity restriction being the most common. School absenteeism data were not available in NFBC1986.

### Socioeconomic patterns of spinal pain

**Fig. 1:**
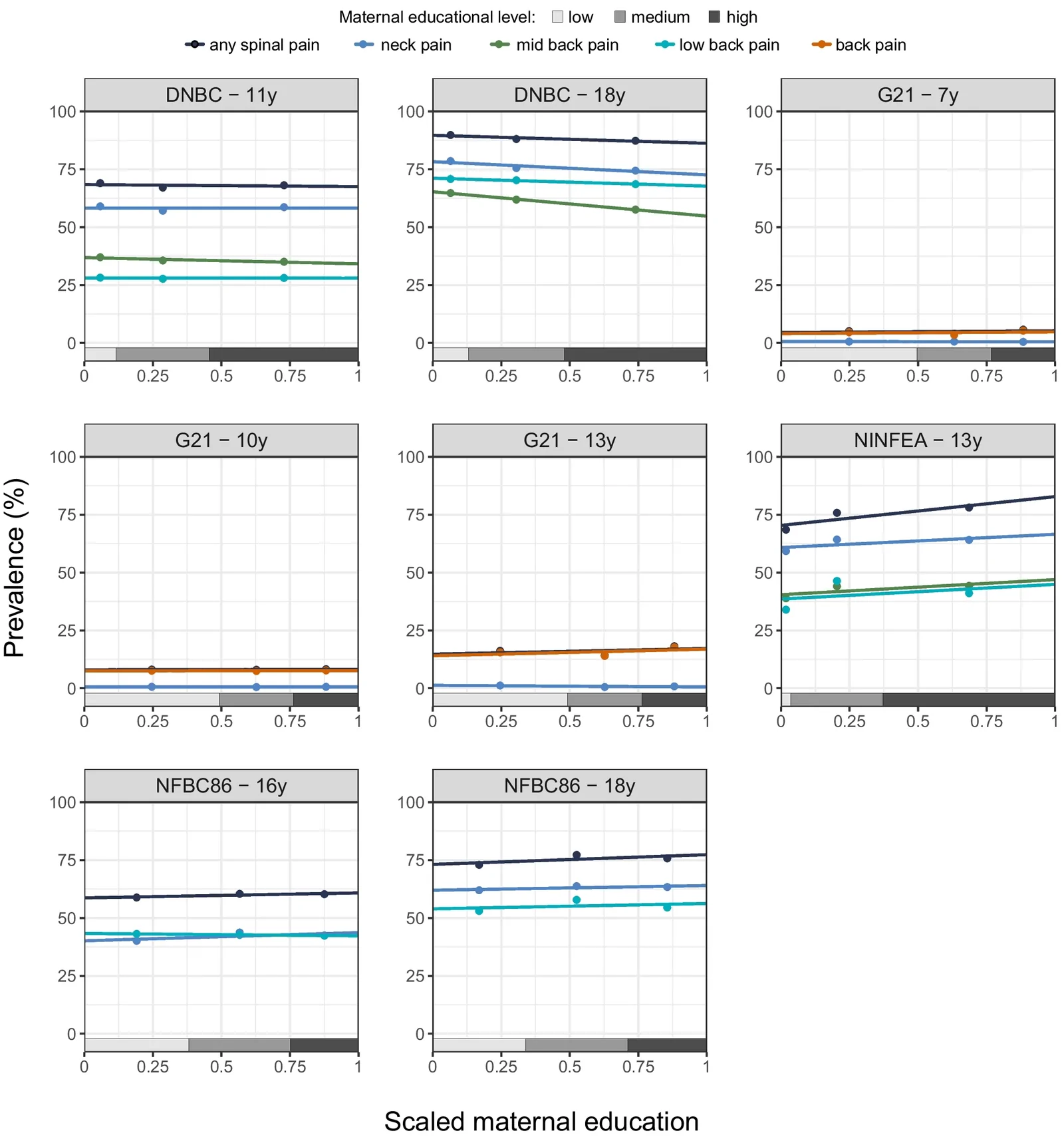
Prevalence of spinal pain at each level of scaled maternal education. The slope of each trendline defines the Slope Index of Inequality (i.e. is given as the difference between the prevalence predicted by the trendline at scaled maternal education = 1 and the prevalence predicted by the trendline at scaled maternal education = 0). The ratio between the prevalence predicted by the trendline at scaled maternal education = 1 and the prevalence predicted by the trendline at scaled maternal education = 0 defines the Relative Index of Inequality. The trendline is obtained by fitting an unadjusted linear regression model to each three data points representing the prevalence of an outcome at each level of scaled maternal education

Figure 1 shows the socioeconomic patterns of spinal pain by anatomical location, illustrated by the linear trendlines showing the prevalence of spinal pain at each level of scaled maternal education. At the DNBC 11-year follow-up, socioeconomic inequalities were generally small, with the largest negative gradient observed for mid-back pain, indicating a higher prevalence among children of mothers with lower education. At age 18, negative gradients were observed for all spinal pain outcomes, with the largest absolute difference for mid-back pain. In G21, socioeconomic gradients were generally small, with negative gradients observed only for neck pain. In contrast, NINFEA showed positive gradients across all anatomical locations, indicating higher prevalences among children of mothers with higher education. In NFBC1986, gradients were also predominantly positive, although generally weaker than those observed in NINFEA, with the exception of low-back pain at age 16, which showed a negative gradient. All SII and RII estimates across spinal pain anatomical locations are shown in Table 3.

**Table 3:** Slope and Relative Index of Inequality (SII and RII, respectively) with 95% confidence intervals of spinal pain at each follow-up in the four participating cohorts

		DNBC		G21			NINFEA	NFBC1986	
Follow-up		11y	18y	7y	10y	13y	13y	16y	18y
Any spinal pain (yes)	SII	− 0.88 [− 6.16, 4.41]	− 3.45 [− 6.26, − 0.65]	0.67 [− 4.61, 5.95]	0.22 [− 0.39, 0.84]	2.46 [− 8.63, 13.54]	12.43 [− 2.25, 27.11]	2.16 [− 0.46, 4.78]	4.19 [− 5.18, 13.56]
	RII	0.99 [0.91, 1.06]	0.96 [0.93, 0.99]	1.15 [− 0.12, 2.41]	1.03 [0.95, 1.11]	1.17 [0.34, 1.99]	1.18 [0.96, 1.40]	1.04 [0.99, 1.08]	1.06 [0.93, 1.19]
Neck pain (yes)	SII	0.02 [− 5.75, 5.78]	− 5.68 [− 10.73, − 0.64]	− 0.16 [− 0.46, 0.14]	− 0.04 [− 0.35, 0.27]	− 0.67 [− 2.14, 0.80]	5.72 [− 5.89, 17.34]	3.54 [− 4.17, 11.24]	2.03 [− 1.46, 5.53]
	RII	1.00 [0.90, 1.10]	0.93 [0.86, 0.99]	0.74 [0.33, 1.16]	0.94 [0.47, 1.40]	0.47 [− 0.35, 1.28]	1.09 [0.90, 1.29]	1.09 [0.89, 1.29]	1.03 [0.98, 1.09]
Mid-back pain (yes)	SII	− 2.68 [− 5.06, − 0.31]	− 10.51 [− 11.56, − 9.47]	n/a	n/a	n/a	6.51 [− 5.26, 18.29]	n/a	n/a
	RII	0.93 [0.86, 0.99]	0.84 [0.82, 0.85]	n/a	n/a	n/a	1.16 [0.86, 1.47]	n/a	n/a
Low-back pain (yes)	SII	− 0.06 [− 1.58, 1.46]	− 3.41 [− 4.24, − 2.58]	n/a	n/a	n/a	6.34 [− 26.86, 39.53]	− 1.03 [− 1.10, − 0.96]	2.29 [− 10.91, 15.49]
	RII	1.00 [0.94, 1.05]	0.95 [0.94, 0.96]	n/a	n/a	n/a	1.16 [0.26, 2.07]	0.98 [0.97, 0.98]	1.04 [0.79, 1.29]
Back pain (yes)	SII	n/a	n/a	0.73 [− 4.73, 6.18]	0.12 [− 0.57, 0.81]	2.85 [− 7.17, 12.86]	n/a	n/a	n/a
	RII	n/a	n/a	1.18 [− 0.32, 2.68]	1.02 [0.92, 1.11]	1.20 [0.41, 2.00]	n/a	n/a	n/a

*n/a*, not available

G21 has a composite ‘back pain’ variable of either mid- or low-back pain

### Socioeconomic patterns of spinal pain frequency, intensity, and functional limitations

**Fig. 2:**
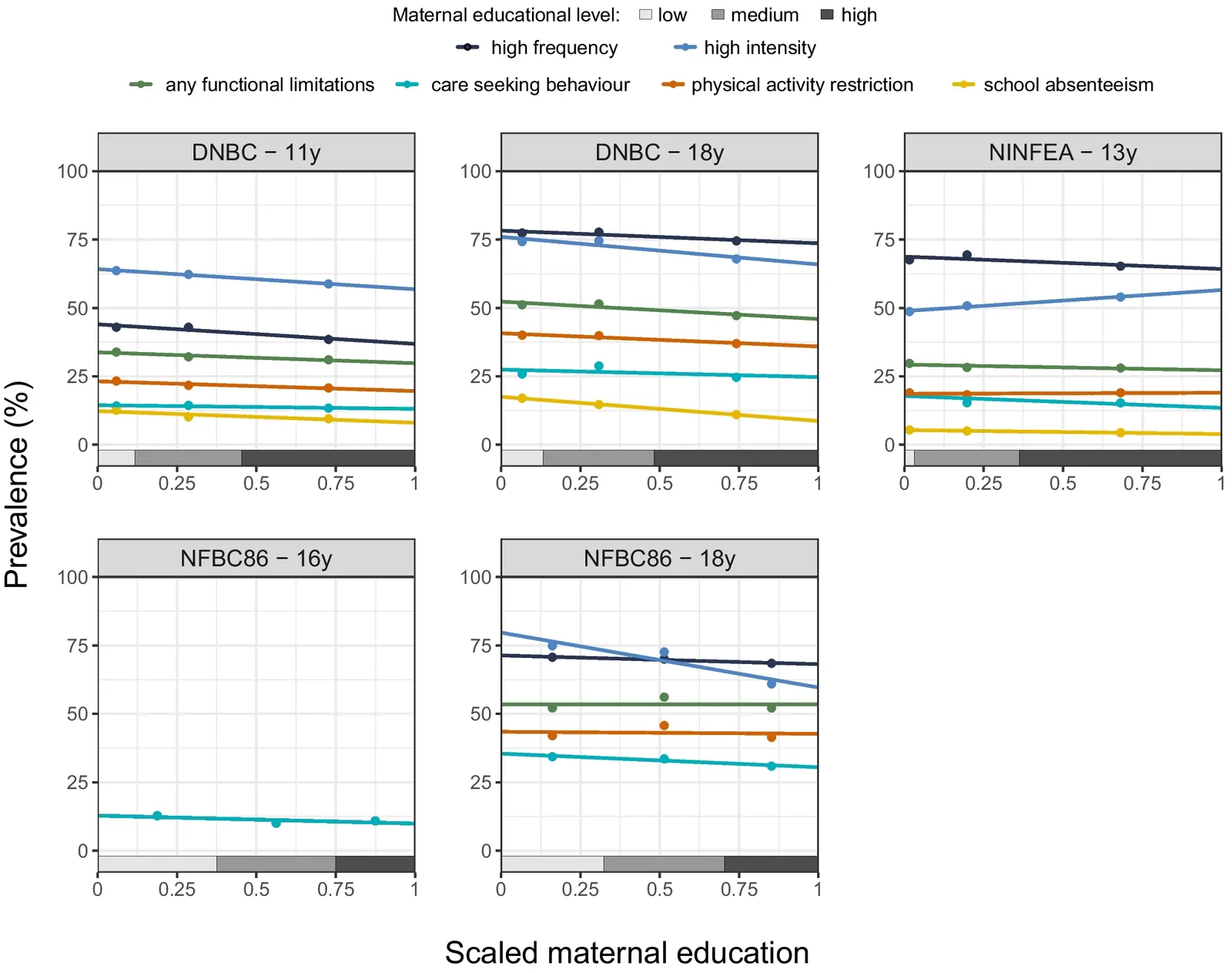
Prevalence of spinal pain high frequency, high intensity, and functional limitations at each level of scaled maternal education. The slope of each trendline defines the Slope Index of Inequality (i.e. is given as the difference between the prevalence predicted by the trendline at scaled maternal education = 1 and the prevalence predicted by the trendline at scaled maternal education = 0). The ratio between the prevalence predicted by the trendline at scaled maternal education = 1 and the prevalence predicted by the trendline at scaled maternal education = 0 defines the Relative Index of Inequality. The trendline is obtained by fitting an unadjusted linear regression model to each three data points representing the prevalence of an outcome at each level of scaled maternal education

Figure 2 shows the socioeconomic patterns of spinal pain frequency, intensity, and functional limitations among children who reported any spinal pain. In DNBC, negative gradients were observed for both high-frequency and high-intensity pain at ages 11 and 18, indicating that children of mothers with lower education had a higher prevalence of frequent or intense pain. In NINFEA, the gradient for high frequency was negative, whereas high intensity showed a positive gradient. In NFBC1986 at age 18, both high frequency and high intensity displayed negative gradients, with a steeper gradient for high intensity. Socioeconomic patterns were not evaluated in G21 because of the small number of children with relevant data.

For pain-related functional limitations, DNBC showed negative gradients for all types of limitations at both follow-ups, generally stronger at age 18 than at age 11, with the steepest gradient observed for school absenteeism at age 18. In NINFEA and NFBC1986, gradients varied, with both positive and negative directions. The strongest negative gradients in both cohorts were observed for care-seeking behaviour. All SII and RII estimates for high frequency, high intensity, and all functional limitations are presented in Table 4.

**Table 4:** Slope and Relative Index of Inequality (SII and RII, respectively) with 95% confidence intervals of spinal pain frequency, intensity and functional limitations at each follow-up in the three^a^ participating cohorts

		DNBC		NINFEA	NFBC1986	
Follow-up		11 y	18 y	13 y	16 y	18 y
High frequency (yes)	SII	− 7.15 [− 12.10, − 2.21]	− 4.63 [− 9.07, − 0.19]	− 4.53 [− 12.35, 3.29]	n/a	− 3.20 [− 4.66, − 1.74]
	RII	0.84 [0.73, 0.94]	0.94 [0.89, 1.00]	0.93 [0.82, 1.05]	n/a	0.96 [0.94, 0.98]
High intensity (yes)	SII	− 7.36 [− 8.24, − 6.48]	− 10.10 [− 18.65, − 1.54]	7.71 [5.50, 9.92]	n/a	− 20.04 [− 35.89, − 4.20]
	RII	0.89 [0.87, 0.90]	0.87 [0.76, 0.97]	1.16 [1.11, 1.21]	n/a	0.75 [0.57, 0.92]
Any functional limitations (yes)	SII	− 3.98 [− 6.64, − 1.33]	− 6.37 [− 12.07, − 0.67]	− 2.09 [− 5.40, 1.21]	− 2.91 [− 8.50, 2.69]	0.10 [− 13.00, 13.20]
	RII	0.88 [0.81, 0.96]	0.88 [0.77, 0.98]	0.93 [0.82, 1.04]	0.77 [0.39, 1.16]	1.00 [0.76, 1.25]
Care seeking behaviour (yes)	SII	− 1.34 [− 2.98, 0.31]	− 2.72 [− 13.69, 8.25]	− 4.35 [− 12.57, 3.86]	− 2.91 [− 8.50, 2.69]	− 4.98 [− 8.21, − 1.74]
	RII	0.91 [0.80, 1.02]	0.90 [0.52, 1.29]	0.76 [0.33, 1.18]	0.77 [0.39, 1.16]	0.86 [0.77, 0.94]
Physical activity restriction (yes)	SII	− 3.58 [− 6.00, − 1.16]	− 4.84 [− 7.80, − 1.88]	0.39 [− 1.79, 2.56]	n/a	− 0.77 [− 14.01, 12.47]
	RII	0.85 [0.75, 0.94]	0.88 [0.81, 0.95]	1.02 [0.90, 1.14]	n/a	0.98 [0.68, 1.28]
School absenteeism (yes)	SII	− 4.26 [− 8.80, 0.28]	− 8.84 [− 9.41, − 8.26]	− 1.48 [− 1.84, − 1.11]	n/a	n/a
	RII	0.65 [0.33, 0.98]	0.50 [0.47, 0.52]	0.73 [0.66, 0.79]	n/a	n/a

*n/a*, not available.

^a^G21 was not included because the number of children with available data on pain frequency, intensity, and functional limitations was insufficient for socioeconomic analyses

## Discussion

In this multicohort study, we observed substantial variation in the prevalence and socioeconomic patterning of spinal pain across four European child and adolescent cohorts. Consistent with previous literature, spinal pain prevalence increased with age, particularly for mid- and low-back pain, and was generally higher among females. Socioeconomic inequalities in spinal pain prevalence were inconsistent and generally modest, whereas clearer socioeconomic differences were observed among children reporting spinal pain, particularly for pain frequency, intensity, and functional limitations.

When interpreting these cross-country differences, methodological heterogeneity across cohorts should be considered. Variation in recall periods, question phrasing, response scales, and age at assessment may affect the sensitivity of spinal pain measures and could lead to differential reporting across socioeconomic groups. For example, shorter recall periods may attenuate socioeconomic gradients, while broader pain definitions or parent-reported measures may amplify them. Similarly, differences in questionnaire framing or mode of data collection may interact with cultural or educational factors, potentially influencing reporting patterns across countries. Despite harmonisation, some residual methodological heterogeneity remained and may contribute to variation in the observed patterns.

### Prevalence differences across cohorts

The wide variation in prevalence, ranging from 4.6% among 7-year-old Portuguese children to 92.3% among 18-year-old Danish adolescents, likely reflects both population differences and methodological heterogeneity [11–15]. Differences in pain reporting, instruments, recall periods, response options, and age at assessment limit direct comparison across cohorts [17, 47–50]. These differences should therefore not necessarily be interpreted as differences in the underlying burden of spinal pain. For example, the prevalence of any spinal pain in NINFEA at age 13 was similar to that in DNBC at age 11, potentially reflecting the use of the same questionnaire and lifetime recall period, despite being conducted in different populations. In contrast, the substantially lower prevalence in G21 may partly reflect its binary pain question, which differed from the graded response options used in the other cohorts. Such measurement differences may have reduced the comparability of prevalence estimates across cohorts.

Pain is inherently subjective, and harmonisation across different instruments cannot eliminate all measurement differences. Our approach classified children as having spinal pain if they reported pain at least once during the specified recall period, enabling a common outcome definition but not distinguishing occasional or mild pain from more clinically meaningful pain. We therefore also examined pain frequency, intensity, and functional limitations to provide a more informative picture of the burden of spinal pain [12].

### Age and sex differences

Spinal pain prevalence generally increased with age, consistent with previous studies [19, 51]. Females generally had higher prevalence of spinal pain than males, with particularly pronounced differences at older ages in DNBC and NFBC1986. Previous studies have reported both no sex differences [25, 52], and higher prevalence among females [12, 13]. The increasing sex difference with age may reflect developmental or psychosocial factors, although these mechanisms could not be evaluated in the present study.

### Socioeconomic patterns

Previous research on socioeconomic inequalities in youth spinal pain has produced mixed findings, with studies reporting higher prevalence or risk among children from less advantaged backgrounds, no clear socioeconomic differences, or differences that varied across outcomes [13, 25, 26, 30]. Our findings similarly showed no consistent cross-cohort socioeconomic pattern in spinal pain prevalence. Some cohorts showed modest socioeconomic gradients, whereas others showed little or no gradient. In contrast, socioeconomic differences were more apparent for pain frequency, intensity, and functional limitations. In DNBC, negative gradients were observed consistently across these outcomes, including particularly pronounced gradients for high-intensity pain and functional limitations at age 18. NFBC1986 also showed negative gradients for high-frequency and high-intensity pain at age 18, whereas NINFEA showed more mixed patterns, including a positive gradient for high-intensity pain. Thus, socioeconomic inequalities appeared more evident in the characteristics and functional impact of spinal pain than in its overall occurrence, although their direction and magnitude varied across cohorts.

### Possible mechanisms

Several plausible pathways may contribute to socioeconomic differences in spinal pain severity and functional limitations. Children from lower-educated families may have greater exposure to lifestyle and environmental factors, including obesity, sedentary behaviour, and reduced access to structured physical activity or organised sports [53, 54]. Consistent with this, recent multinational evidence indicates that moderate-to-vigorous physical activity is associated with modestly lower odds of backache among children and adolescents [55].

Psychosocial stressors, including family financial strain and limited social support, may influence pain perception, coping, and functional limitations [20]. Differences in health literacy and care-seeking behaviour may also contribute to socioeconomic differences in pain impact; for example, families with higher education may be more likely to seek early intervention. Genetic and early-life factors, including prenatal exposures, birth outcomes, and early growth trajectories, may interact with socioeconomic circumstances to influence vulnerability to severe or persistent spinal pain. Evidence for such early-life effects is mixed. For example, a Danish cohort study found that girls born very preterm were slightly less likely to report severe spinal pain in pre-adolescence, with no association observed among boys [56]. Together, these pathways may provide possible explanations for why socioeconomic differences were more apparent in pain severity and functional impact than in overall prevalence, but our study did not assess these mechanisms and therefore cannot determine which mechanisms underlie the observed socioeconomic patterns.

## Strengths and limitations

A major strength of this study is the use of federated individual-level data, which enabled cross-cohort comparisons without sharing raw data. Harmonisation across four European cohorts allowed socioeconomic patterns to be examined across different sociocultural contexts.

Several limitations should be considered. First, differences in questionnaires, recall periods, response options, and data collection across cohorts may have affected prevalence estimates and socioeconomic gradients despite harmonisation. Second, selection bias cannot be excluded because information on non-responders was unavailable and inverse probability weighting could not be applied. Missing data also varied across cohorts and outcomes. Third, the participating cohorts represent birth years spanning several decades, introducing potential cohort effects. Fourth, SII and RII estimation assumes linearity, and estimates may be misleading if a straight line does not adequately capture the underlying socioeconomic pattern. Confidence intervals in such cases are also wide. In addition, when prevalence approaches ceiling levels in certain subgroups, the RII may be difficult to interpret because relative differences are compressed and small absolute variations can produce unstable estimates. Fifth, as this was a descriptive study aimed at quantifying socioeconomic inequalities rather than estimating causal effects, potential confounders were not included in the regression models. The observed associations should consequently not be interpreted as causal effects of maternal education. Sixth, maternal education was the only indicator of socioeconomic background available across all cohorts and does not fully capture socioeconomic conditions. Additional indicators such as parental occupation, income, and paternal education would provide a more comprehensive assessment. Seventh, maternal education was harmonised into only three categories. Although this ensured comparability, the relatively coarse categorisation may have reduced sensitivity to subtle socioeconomic differences and attenuated inequality estimates, particularly where associations were non-linear. Finally, we lacked information on potential mechanisms such as sports participation, trauma, physical inactivity, or menstruation, which may help explain variation in prevalence and socioeconomic patterns.

## Clinical implications

Although absolute socioeconomic differences were generally modest, differences in pain intensity and functional limitations may be relevant at the population level given the high prevalence of spinal pain during adolescence. Children with more severe or disabling pain may be at greater risk of persistent symptoms and reduced participation in daily activities. The findings suggest that attention to pain severity and its impact on daily functioning may be particularly important among children from less advantaged backgrounds. Prevention strategies should therefore reduce the overall burden of spinal pain, while targeted support for children with more severe or disabling pain may help reduce inequalities in its impact.

## Future research

Future research should investigate mechanisms linking socioeconomic disadvantage to the frequency, intensity, and functional impact of spinal pain, including psychosocial stress, sleep, physical activity, and mental health. Cross-cohort longitudinal studies using harmonised measures could improve understanding of developmental trajectories and cross-national differences. Intervention studies are also needed to determine whether school‑ or community-based programmes can reduce both the burden of spinal pain and inequalities in its impact.

## Conclusion

This study identified substantial heterogeneity in socioeconomic patterns of spinal pain across European child and adolescent cohorts. Inequalities in spinal pain prevalence were inconsistent and generally modest, whereas socioeconomic differences were more apparent in pain frequency, intensity, and functional limitations, particularly in DNBC and NFBC1986. However, the direction and magnitude of these differences varied across settings. These findings suggest that socioeconomic inequalities may be more evident in the severity and functional consequences of spinal pain than in its overall occurrence. Spinal pain was prevalent, increased with age, and was consistently more common among females, underscoring the importance of preventive efforts addressing both the overall burden of spinal pain and inequalities in its impact. Future research using harmonised and comprehensive pain assessments is needed to clarify the mechanisms underlying these diverse socioeconomic patterns.

## Supplementary Information

Below is the link to the electronic supplementary material.

Supplementary file1 (DOCX 484 kb)

## Data Availability

This study used harmonised data from four birth cohorts participating in the EU Child Cohort Network (ECCN). Data are not publicly available; access requires a proposal to the ECCN committee and subsequent approval by individual cohorts, after which accredited researchers are granted remote access via DataSHIELD, ensuring no individual-level data leave host sites. For data access enquiries, contact the coordinating centre (EU.Child.Cohort@erasmusmc.nl); for cohort-specific data sharing policies, contact the respective cohorts.

## Abbreviations

DNBC: Danish National Birth Cohort
G21: Generation 21
NINFEA: Nascita e INFanzia: gli Effetti dell’Ambiente
NFBC1986: Northern Finland Birth Cohort 1986
SII: Slope Index of Inequality
RII: Relative Index of Inequality
YSQ: Young Spine Questionnaire
LPQ: Luebeck Pain Questionnaire
ISCED: International Standard Classification of Education
