# Supplementary material for: Spinal pain prevalence in childhood and socioeconomic patterns: a comparative study in four European birth cohorts
Source: Eur J Pediatr. 2026 Oct 3;185(11):800. doi: 10.1007/s00431-026-07461-w (PMC13633969; doi:10.1007/s00431-026-07461-w)
Supplement: Supplementary file 1 [file 431_2026_7461_MOESM1_ESM.docx]

**Supplementary material**

**Supplementary A: EU Child Cohort Network and participating cohorts**

The EU Child Cohort Network is a collaborative infrastructure established within the EU‑funded LifeCycle Project. It harmonises data from large European pregnancy and birth cohorts and enables federated, cross‑cohort analyses through standardised variable definitions, shared governance procedures, and privacy‑preserving analytical tools. Cohorts contribute harmonised variables to secure local servers, allowing analyses to be performed without transferring individual‑level data.

The Danish National Birth Cohort (DNBC, Denmark) is a population-based birth cohort including mothers and their children born between 1996 and 2003, with multiple follow-ups from pregnancy through childhood and young adulthood [1; 2]. The Geração 21 (G21, Portugal) cohort includes mothers and their children born between April 2005 and August 2006, with multiple follow-ups from birth onwards [3]. The Nascita e INFanzia: gli Effetti dell’Ambiente (NINFEA, Italy) is an internet-based birth cohort that recruited pregnant women between 2005 and 2016 and follows children through multiple follow-ups across childhood, adolescence, and early adulthood [4]. The NINFEA database version 2024.01 was used in this study. The Northern Finland Birth Cohort 1986 (NFBC1986, Finland) includes children born in Northern Finland between 1 July 1985 and 30 June 1986 [5-8].

**Supplementary B: Questions on spinal pain per cohort and follow-up**

The following tables present the spinal pain questions used in each cohort and follow‑up. For each item, the distribution of responses (counts and percentages) prior to harmonisation is also provided in red, with percentages calculated using the number of participants who responded to the respective question as the denominator. For data protection, counts for less than 3 individuals are not reported and are indicated as “n/d” (not disclosed). For those cases, the percentage of the corresponding complementary categories are also not disclosed.

**DNBC 11-year follow-up**

| E089: Have you suffered from neck pain | 1: yes, often | 2: yes, from time to time | 3: once or twice | 4: never |
| --- | --- | --- | --- | --- |
|  | 1573 (3.3%) | 8171 (17.2%) | 17896 (37.8%) | 19741 (41.7%) |

| E089A (neck): How much did it hurt at its worst? 1 is "not at all" and 6 is "really very much”. | 1: 1 | 2: 2 | 3: 3 | 4: 4 | 5: 5 | 6: 6 |
| --- | --- | --- | --- | --- | --- | --- |
|  | 3431 (12.5%) | 9199 (33.4%) | 8216 (29.8%) | 4484 (16.3%) | 1610  (5.8%) | 611  (2.2%) |

| E090: Have you suffered from middle back pain? | 1: yes, often | 2: yes, from time to time | 3: once or twice | 4: never |
| --- | --- | --- | --- | --- |
|  | 888 (1.9%) | 4117 (8.7%) | 11957 (25.3%) | 30389 (64.2%) |

| E090A (mid back): How much did it hurt at its worst? 1 is "not at all" and 6 is "really very much”. | 1: 1 | 2: 2 | 3: 3 | 4: 4 | 5: 5 | 6: 6 |
| --- | --- | --- | --- | --- | --- | --- |
|  | 2574 (15.3%) | 5888 (34.9%) | 4650 (27.6%) | 2453 (14.5%) | 970  (5.8%) | 333  (2.0%) |

| E091: Have you suffered from low back pain? | 1: yes, often | 2: yes, from time to time | 3: once or twice | 4: never |
| --- | --- | --- | --- | --- |
|  | 783 (1.7%) | 3098 (6.5%) | 9573 (20.2%) | 33897 (71.6%) |

| E091A (low back): How much did it hurt at its worst? 1 is "not at all" and 6 is "really very much”. | 1: 1 | 2: 2 | 3: 3 | 4: 4 | 5: 5 | 6: 6 |
| --- | --- | --- | --- | --- | --- | --- |
|  | 2211 (16.5%) | 4553 (34.0%) | 3371 (25.2%) | 1983 (14.8%) | 896  (6.7%) | 361  (2.7%) |

| E092: Have you stayed home from school because of neck or back pain? | 1: yes, often | 2: yes, from time to time | 3: once or twice | 4: never |
| --- | --- | --- | --- | --- |
|  | 55 (0.1%) | 468 (1.0%) | 3018 (6.4%) | 43798 (92.5%) |

| E093: Has neck or back pain stopped you from doing sports? | 1: yes, often | 2: yes, from time to time | 3: once or twice | 4: never |
| --- | --- | --- | --- | --- |
|  | 235 (0.5%) | 1985 (4.1%) | 6117 (12.7%) | 39901 (82.7%) |

| E094: Have you seen a doctor, a chiropractor or physiotherapist for your neck or back pain? | 1: yes, often | 2: yes, from time to time | 3: once or twice | 4: never |
| --- | --- | --- | --- | --- |
|  | 479 (1.0%) | 1138 (2.4%) | 3157 (6.7%) | 42564 (89.9%) |

**DNBC 18-year follow-up**

| G128: Have you within the last year suffered from neck pain? | 4: don’t know | 3: yes, often | 2: yes, occasionally | 1: yes, once or twice | 0: no |
| --- | --- | --- | --- | --- | --- |
|  | 677 (1.5%) | 7849 (17.4%) | 12922 (28.7%) | 12639 (28.1%) | 10948 (24.3%) |

| G129 (neck): How much did it hurt when it hurt the most? 0 is “it did not hurt” and 10 is “it hurt very much” | 0: 0 | 1: 1 | 2: 2 | 3: 3 | 4: 4 | 5: 5 | 6: 6 | 7: 7 | 8: 8 | 9: 9 | 10: 10 |
| --- | --- | --- | --- | --- | --- | --- | --- | --- | --- | --- | --- |
|  | 98 (0.3%) | 1978 (5.9%) | 5684 (17.0%) | 7291 (21.8%) | 5306 (15.9%) | 4557  (13.6%) | 3619 (10.8%) | 2387 (7.1%) | 1523 (4.6%) | 583 (1.7%) | 384 (1.1%) |

| G130: Have you within the last year suffered from middle back pain? | 4: don’t know | 3: yes, often | 2: yes, occasionally | 1: yes, once or twice | 0: no |
| --- | --- | --- | --- | --- | --- |
|  | 1095 (2.4%) | 5730 (12.7%) | 10355 (23.0%) | 10288 (22.8%) | 17567 (39.0%) |

| G131 (mid back): How much did it hurt when it hurt the most? 0 is “it did not hurt” and 10 is “it hurt very much” | 0: 0 | 1: 1 | 2: 2 | 3: 3 | 4: 4 | 5: 5 | 6: 6 | 7: 7 | 8: 8 | 9: 9 | 10: 10 |
| --- | --- | --- | --- | --- | --- | --- | --- | --- | --- | --- | --- |
|  | 61 (0.2%) | 1315 (5.0%) | 3856  (14.6%) | 4967 (18.8%) | 4516 (17.1%) | 4025 (15.3%) | 3048 (11.6%) | 2145 (8.1%) | 1421 (5.4%) | 597 (2.3%) | 422 (1.6%) |

| G132: Have you within the last year suffered from lower back pain? | 4: don’t know | 3: yes, often | 2: yes, occasionally | 1: yes, once or twice | 0: no |
| --- | --- | --- | --- | --- | --- |
|  | 931 (2.1%) | 8426 (18.7%) | 12466 (27.7%) | 9728 (21.6%) | 13484 (29.9%) |

| G133 (low back): How much did it hurt when it hurt the most? 0 is “it did not hurt” and 10 is “it hurt very much” | 0: 0 | 1: 1 | 2: 2 | 3: 3 | 4: 4 | 5: 5 | 6: 6 | 7: 7 | 8: 8 | 9: 9 | 10: 10 |
| --- | --- | --- | --- | --- | --- | --- | --- | --- | --- | --- | --- |
|  | 62 (0.2%) | 1417 (4.6%) | 3777 (12.3%) | 5271  (17.2%) | 4705  (15.4%) | 4510 (14.7%) | 3793 (12.4%) | 3083 (10.1%) | 2193 (7.2%) | 1038 (3.4%) | 771 (2.5%) |

| G134_1: Within the last year, has pain in the neck, (upper) back or lower back… caused you to stay home from school/work? | 4: don’t know | 3: yes, often | 2: yes, occasionally | 1: yes, once or twice | 0: no |
| --- | --- | --- | --- | --- | --- |
|  | 122 (0.3%) | 308 (0.8%) | 1191 (3.0%) | 3764 (9.6%) | 33872 (86.3%) |

| G134_2: Within the last year, has pain in the neck, (upper) back or lower back… prohibited you from doing sports/exercise? | 4: don’t know | 3: yes, often | 2: yes, occasionally | 1: yes, once or twice | 0: no |
| --- | --- | --- | --- | --- | --- |
|  | 413 (1.1%) | 1567 (4.0%) | 4143 (10.6%) | 9259 (23.6%) | 23875 (60.8%) |

| G134_3: Within the last year, has pain in the neck, (upper) back or lower back… made you see a doctor, acupuncturist, chiropractor, or physiotherapist? | 4: don’t know | 3: yes, often | 2: yes, occasionally | 1: yes, once or twice | 0: no |
| --- | --- | --- | --- | --- | --- |
|  | 97 (0.2%) | 2207 (5.6%) | 3251 (8.3%) | 4878 (12.4%) | 28824 (73.4%) |

**G21 7-, 10-, and 13-year follow-ups**

| In the last three months, did [participant] have any pain? | 1: Yes | 0: No |
| --- | --- | --- |
| 7-year follow-up (parent-reported) | 2601 (44.7%) | 3212 (55.3%) |
| 10-year follow-up (parent-reported) | 3131 (58.5%) | 2221 (41.5%) |
| 13-year follow-up (self-reported) | 2645 (57.9%) | 1921 (42.1%) |
| If yes, then:  A3: Select all the sites where [participant] felt pain in the last 3 months* | Responses selected from 10 sites (head, back, ears, abdomen, pelvis, arms, legs, chest, throat, teeth) and/or specified in an open field. All responses were examined and recoded, as appropriate, to isolate the spinal pain regions used in this study. | |
| 7-year follow-up (parent-reported) | Back pain: 257 (9.9%); Neck pain: 31 (1.2%) | |
| 10-year follow-up (parent-reported) | Back pain: 406 (13.0%); Neck pain: 34 (1.1%) | |
| 13-year follow-up (self-reported) | Back pain: 711 (26.9%); Neck pain: 44 (1.7%) | |

***** This was preceded by the question: In the last three months, did [participant] have any pain?

| A6: In the last 3 months, how often has [participant] had this pain (site marked as principal or most bothersome from all the sites selected)? | 4: every day | 3: more than once a week but not every day | 2: 1 time per week | 1: 1 to 3 times a month | 0: Less than once a month |
| --- | --- | --- | --- | --- | --- |
| 7-year follow-up (parent-reported) | 7 (11.7%) | 8 (13.3%) | 3 (5.0%) | 11 (18.3%) | 31 (51.7%) |
| 10-year follow-up (parent-reported) | 8 (6.4%) | 16 (12.8%) | 10 (8.0%) | 27 (21.6%) | 64 (51.2%) |
| 13-year follow-up (self-reported) | 37 (12.3%) | 58 (19.3%) | 40 (13.3%) | 61 (20.3%) | 104 (34.7%) |

| A7: In general, what was the intensity of this pain (site marked as principal or most bothersome from all the sites selected)* in the last 3 months? | Intensity slider from ‘minimal pain’ to ‘most severe pain possible’** | | | | | | | | | | |
| --- | --- | --- | --- | --- | --- | --- | --- | --- | --- | --- | --- |
|  | [1-5[ :0 | [5-15[ : 1 | [15-25[ : 2 | [25-35[ : 3 | [35-45[ : 4 | [45-55[ : 5 | [55-65[ : 6 | [65-75[ : 7 | [75-85[ : 8 | [85-95[ : 9 | [95-100] : 10 |
| 7-year follow-up (parent-reported) | 3 | 3 | n/d | 15 | n/d | 3 | n/d | n/d | n/d | n/d | n/d |
| 10-year follow-up (parent-reported) | n/d | 7 | 18 | 7 | 5 | 12 | 6 | 7 | n/d | n/d | n/d |
| 13-year follow-up (self-reported) | 4 | 8 | 15 | 16 | 18 | 52 | 31 | 34 | 25 | 4 | n/d |

***** For this study, only G21 cases where the back or neck regions were indicated as the principal or most bothersome pain sites are included. Additionally, the G21 questionnaire did not allow reporting on midback and lower back pain separately, and pain intensity was only queried when the principal or most bothersome pain was reported to have occurred more than once in the last three months.

** This split of values is based on the intensity slider data on a scale from 1-100mm (a 100-point scale), which is converted to an 11-point scale ranging from 0 to 10.

| A9: Due to this pain (site marked as principal or most bothersome from all the sites selected)*, [participant]…  - Went to a doctor | 1: selected “went to a doctor” | 0: did not select “went to a doctor” |
| --- | --- | --- |
| 7-year follow-up (parent-reported) | 11 (31.4%) | 24 (68.6%) |
| 10-year follow-up (parent-reported) | 26 (40.0%) | 39 (60.0%) |
| 13-year follow-up (self-reported) | Not included in G21 13-year questionnaire | Not included in G21 13-year questionnaire |

***** For this study, only G21 cases where the back or neck regions were indicated as the principal or most bothersome pain sites are included. Additionally, the G21 questionnaire did not allow reporting on midback and lower back pain separately, and consequences of pain were only queried when the principal or most bothersome pain was reported to have occurred more than once in the last three months.

| A9: Due to this pain (site marked as principal or most bothersome from all the sites selected), [participant]… Was unable to do their free time activities | 1: selected “was unable to do their free time activities” | 0: did not select “was unable to do their free time activities” |
| --- | --- | --- |
| 7-year follow-up (parent-reported) | 5 (14.3%) | 30 (85.7%) |
| 10-year follow-up (parent-reported) | n/d | 64 |
| 13-year follow-up (self-reported) | 38 (18.0%) | 173 (82.0%) |

| A9: Due to this pain (site marked as principal or most bothersome from all the sites selected), [participant]… Missed classes | 1: selected “missed classes” | 0: did not select “missed classes” |
| --- | --- | --- |
| 7-year follow-up (parent-reported) | 3 (8.6%) | 32 (91.4%) |
| 10-year follow-up (parent-reported) | 7 (10.8%) | 58 (89.2%) |
| 13-year follow-up (self-reported) | 15 (7.1%) | 196 (92.9%) |

**NINFEA 13-year follow-up**

Note that the NINFEA 13-year follow-up is still ongoing. These numbers are based on the data version 2024.01 (January 2024). At that time, only a subset of the cohort (children with at least 13 years of age, n~3100) was eligible to participate in the 13-year follow-up.

| N1: How often have you had neck pain? | 3: often | 2: once in a while | 1: once or twice | 0: never |
| --- | --- | --- | --- | --- |
|  | 71 (4.7%) | 478 (32.0%) | 410 (27.4%) | 537 (35.9%) |

| N2: Click on the face that shows how bad you felt when the pain in your neck was strongest, where 1 is “no pain” and 6 is “very much pain” | 1: 1 | 2: 2 | 3: 3 | 4: 4 | 5: 5 | 6: 6 |
| --- | --- | --- | --- | --- | --- | --- |
|  | 45 (4.7%) | 516 (53.9%) | 251 (26.2%) | 93 (9.7%) | 42 (4.4%) | 10 (1.0%) |

| N3: How often have you had middle back pain? | 3: often | 2: once in a while | 1: once or twice | 0: never |
| --- | --- | --- | --- | --- |
|  | 56 (3.7%) | 292 (19.5%) | 312 (20.8%) | 837 (55.9%) |

| N4: Click on the face that shows how bad you felt when the pain in your middle back was strongest, where 1 is “no pain” and 6 is “very much pain” | 1: 1 | 2: 2 | 3: 3 | 4: 4 | 5: 5 | 6: 6 |
| --- | --- | --- | --- | --- | --- | --- |
|  | 40 | 364 | 169 | 58 | 23 | n/d |

| N5: How often have you had low back pain? | 3: often | 2: once in a while | 1: once or twice | 0: never |
| --- | --- | --- | --- | --- |
|  | 67 (4.5%) | 289 (19.3%) | 280 (18.7%) | 858 (57.4%) |

| N6: Click on the face that shows how bad you felt when the pain in your low back was strongest, where 1 is “no pain” and 6 is “very much pain” | 1: 1 | 2: 2 | 3: 3 | 4: 4 | 5: 5 | 6: 6 |
| --- | --- | --- | --- | --- | --- | --- |
|  | 28 (4.4%) | 312 (49.3%) | 186 (29.4%) | 73 (11.5%) | 30 (4.7%) | 4 (0.6%) |

| N7: Have you stayed home from school because of neck or back pain? | 3: often | 2: once in a while | 1: once or twice | 0: never |
| --- | --- | --- | --- | --- |
|  | n/d | 7 | 47 | 1443 |

| N8: Has neck or back pain stopped you from doing sports? | 3: often | 2: once in a while | 1: once or twice | 0: never |
| --- | --- | --- | --- | --- |
|  | 5 (0.3%) | 66 (4.4%) | 148 (9.9%) | 1276 (85.4%) |

| N9: Have you been to a doctor, physical therapist, chiropractor because of neck or back pain? | 3: often | 2: once in a while | 1: once or twice | 0: never |
| --- | --- | --- | --- | --- |
|  | 16 (1.1%) | 64 (4.3%) | 102 (6.8%) | 1312 (87.8%) |

**NFBC1986 16-year follow-up**

| 28-neck: Have you had pain or aching during the past six months in the following body parts? | 2: yes, and I have gone to a doctor, physiotherapist, a health nurse etc. because of it | 1: yes, but I haven’t gone to a doctor, physiotherapist, a health nurse etc. because of it | 0: no |
| --- | --- | --- | --- |
|  | 199 (2.8%) | 2755 (39.2%) | 4076 (58.0%) |

| 28-low back: Have you had pain or aching during the past six months in the following body parts? | 2: yes, and I have gone to a doctor, physiotherapist, a health nurse etc. because of it | 1: yes, but I haven’t gone to a doctor, physiotherapist, a health nurse etc. because of it | 0: no |
| --- | --- | --- | --- |
|  | 338 (4.8%) | 2674 (38.1%) | 4011 (57.1%) |

**NFBC1986 18-year follow-up**

| 34-neck: Have you had pain or aching during the past six months in the following body parts? | 2: yes, and I have gone to a doctor, physiotherapist, a health nurse etc. because of it | 1: yes, but I haven’t gone to a doctor, physiotherapist, a health nurse etc. because of it | 0: no |
| --- | --- | --- | --- |
|  | 105 (5.5%) | 1088 (57.1%) | 713 (37.4%) |

| 34-low back: Have you had pain or aching during the past six months in the following body parts? | 2: yes, and I have gone to a doctor, physiotherapist, a health nurse etc. because of it | 1: yes, but I haven’t gone to a doctor, physiotherapist, a health nurse etc. because of it | 0: no |
| --- | --- | --- | --- |
|  | 108 (5.7%) | 945 (49.7%) | 848 (44.6%) |

| 36: Have you ever had low back pain? | 1: yes | 0: no |
| --- | --- | --- |
|  | 1016 (52.7%) | 912 (47.3%) |

| 42: How many days in total have you been having lower back pain during the last year? | 4: every day | 3: more than 30 days, but not everyday | 2: 8-30 days | 1: 1-7 days | 0: 0 days |
| --- | --- | --- | --- | --- | --- |
|  | 21 (2.1%) | 193 (18.9%) | 334 (32.6%) | 399 (39.0%) | 76 (7.4%) |

| 43: How difficult was your worst episode of lower back pain? 0 is “no pain at all” and 10 is “worst possible pain” | 0: 0 | 1: 1 | 2: 2 | 3: 3 | 4: 4 | 5: 5 | 6: 6 | 7: 7 | 8: 8 | 9: 9 | 10: 10 |
| --- | --- | --- | --- | --- | --- | --- | --- | --- | --- | --- | --- |
|  | 16 (1.5%) | 41 (4.0%) | 126 (12.2%) | 196  (18.9%) | 163  (15.7%) | 134 (12.9%) | 112 (10.8%) | 115 (11.1%) | 87 (8.4%) | 36 (3.5%) | 9 (0.9%) |

| 45: Have you - because of your lower back pain – had to... | 1: yes | 0: no |
| --- | --- | --- |
| b) To see a doctor? | 171 (16.7%) | 851 (83.3%) |
| c) To see a physiotherapist? | 148 (14.5%) | 875 (85.5%) |
| d) To see a health nurse | 139 (13.6%) | 880 (86.4%) |
| e) To see a chiropractor, naprapath or osteopath | 45 (4.4%) | 971 (95.6%) |
| f) To see another nurse | 51 (5.1%) | 943 (94.9%) |

| 46: Has your lower back pain bothered you in the following functions: j) exercise or sports | 1: yes | 0: no |
| --- | --- | --- |
|  | 445 (43.6%) | 575 (56.4%) |

| 47: Have you - due to your lower back pain - had to reduce exercise or sports? | 2: yes, I had to stop completely | 1: yes, I’ve had to cut back | 0: no |
| --- | --- | --- | --- |
|  | 4 (0.4%) | 152 (14.8%) | 873 (84.8%) |

| 61: Have you ever had neck pain? | 1: yes | 0: no |
| --- | --- | --- |
|  | 1299 (67.2%) | 633 (32.8%) |

| 64: How many days in total have you been having neck pain during the last year? | 4: every day | 3: more than 30 days, but not everyday | 2: 8-30 days | 1: 1-7 days | 0: 0 days |
| --- | --- | --- | --- | --- | --- |
|  | 31 (2.4%) | 342 (26.5%) | 410 (31.7%) | 456 (35.3%) | 53 (4.1%) |

| 65: How difficult was your worst episode of neck pain? 0 is “no pain at all” and 10 is “worst possible pain” | 0: 0 | 1: 1 | 2: 2 | 3: 3 | 4: 4 | 5: 5 | 6: 6 | 7: 7 | 8: 8 | 9: 9 | 10: 10 |
| --- | --- | --- | --- | --- | --- | --- | --- | --- | --- | --- | --- |
|  | 7 (0.5%) | 74 (5.7%) | 209 (16.0%) | 250  (19.1%) | 188  (14.4%) | 185 (14.2%) | 120 (9.2%) | 116 (8.9%) | 84 (6.4%) | 44 (3.4%) | 29 (2.2%) |

| 67: Have you - because of your neck pain – had to... | 1: yes | 0: no |
| --- | --- | --- |
| b) To see a doctor? | 106 (8.2%) | 1187 (91.8%) |
| c) To see a physiotherapist? | 103 (8.0%) | 1190 (92.0%) |
| d) To see a health nurse | 113 (8.8%) | 1177 (91.2%) |
| e) To see a chiropractor, naprapath or osteopath | 23 (1.8%) | 1265 (98.2%) |
| f) To see another nurse | 72 (5.7%) | 1194 (94.3%) |

| 68: Has your neck pain bothered you in the following functions: j) exercise or sports | 1: yes | 0: no |
| --- | --- | --- |
|  | 335 (26.0%) | 955 (74.0%) |

**Supplementary C: Harmonisation of spinal pain variables**

This section describes the procedure used to harmonise spinal pain variables across cohorts. Because the participating cohorts used different instruments, recall periods, and response formats, all spinal pain items were mapped to a common set of ten harmonised variables: pain in three anatomical locations (neck, mid‑back, low‑back), any spinal pain, pain frequency, pain intensity, three specific functional limitations (care seeking behaviour, physical activity restriction, school absenteeism), and any functional limitations.

Authors responsible for harmonising the spinal pain variables were provided with harmonisation instructions, together with the cohort‑specific questionnaire information summarised in Supplementary B. These instructions guided the mapping of original cohort items to the harmonised variables and ensured consistency across cohorts.

Pain frequency was harmonised into a binary variable indicating high frequency, with responses such as ‘once or twice’ classified as no and ‘often’ as yes. Pain intensity was similarly harmonised into a binary variable indicating high intensity, with ‘mild’ or ‘moderate’ pain classified as no and ‘severe’ pain as yes. These cut‑offs were selected to maximise comparability across cohorts with different response formats while preserving meaningful distinctions in symptom severity.

Functional limitation items were coded as binary indicators (yes/no). “Any functional limitations” was defined as reporting at least one of the three limitations: care‑seeking behaviour, physical activity restriction, school absenteeism. Not all variables were available in all cohorts due to differences in questionnaire content. The mapping of original items to harmonised variables is provided below.

| **sp_neck: Have you had any neck pain?** | | | | | | | |
| --- | --- | --- | --- | --- | --- | --- | --- |
| Cohort | DNBC 11y | DNBC 18y | G21 7y, 10y, 13y | NINFEA 13y | NFBC1986 16y | NFBC1986 18y | Values of sp_neck |
| Item | E089; [4] | G128; [0] | A3; [did not select neck pain] | N1; [0] | 28-neck; [0] | 34-neck; [0] | NO = [0] |
|  | E089; [1-3] | G128; [1-3] | A3; [selected neck pain] | N1; [1-3] | 28-neck; [1-2] | 34-neck; [1-2] | YES = [1] |
|  | E089; not answered | G128; [4] OR not answered | A3; not answered | N1; not answered | 28-neck; not answered | 34-neck; not answered | Missing / not answered =  Set as NA |

| **sp_midback: Have you had any mid back pain?** | | | | |
| --- | --- | --- | --- | --- |
| Cohort | DNBC 11y | DNBC 18y | NINFEA 13y | Values of sp_midback |
| Item | E090; [4] | G130; [0] | N3; [0] | NO = [0] |
|  | E090; [1-3] | G130; [1-3] | N3; [1-3] | YES = [1] |
|  | E090; not answered | G130; [4] OR not answered | N3; not answered | Missing/ not answered =  Set as NA |

| **sp_lowback: Have you had any low back pain?** | | | | | | |
| --- | --- | --- | --- | --- | --- | --- |
| Cohort | DNBC 11y | DNBC 18y | NINFEA 13y | NFBC1986 16y | NFBC1986 18y | Values of sp_lowback |
| Item | E091; [4] | G132; [0] | N5; [0] | 28-low back; [0] | 34-low back; [0] | NO = [0] |
|  | E091; [1-3] | G132; [1-3] | N5; [1-3] | 28-low back; [1-2] | 34-low back; [1-2] | YES = [1] |
|  | E091; not answered | G132; [4] OR not answered | N5; not answered | 28-low back; not answered | 34-low back;  not answered | Missing/ not answered =  Set as NA |

| **sp_back: Have you had any back pain?** | | |
| --- | --- | --- |
| Cohort | G21 7y, 10y, 13y | Values of sp_back |
| Item | A3; [did not select back pain] | NO = [0] |
|  | A3; [selected back pain] | YES = [1] |
|  | A3; not answered | Missing/ not answered =  Set as NA |

| **sp_: Have you had any spinal pain?** | | | | | | | |
| --- | --- | --- | --- | --- | --- | --- | --- |
| Cohort | DNBC 11y | DNBC 18y | G21 7y, 10y, 13y | NINFEA 13y | NFBC1986 16y | NFBC1986 18y | Values of sp_ |
| Item | sp_neck; [0] AND sp_midback; [0] AND  sp_lowback; [0] | sp_neck; [0] AND sp_midback; [0] AND  sp_lowback; [0] | sp_neck; [0]  AND  sp_back; [0]  AND  no mentions of spine in the Other region open field | sp_neck; [0] AND sp_midback; [0] AND  sp_lowback; [0] | sp_neck; [0] AND  sp_lowback;[0] | sp_neck; [0] AND  sp_lowback; [0] | NO = [0] |
|  | sp_neck; [1]  OR  sp_midback; [1] OR  sp_lowback; [1] | sp_neck; [1] OR sp_midback; [1] OR  sp_lowback; [1] | sp_neck; [1]  OR  sp_back; [1]  OR  one or more mentions of spine in the Other region open field | sp_neck; [1]  OR  sp_midback; [1] OR  sp_lowback; [1] | sp_neck; [1]  OR  sp_lowback; [1] | sp_neck; [1]  OR  sp_lowback; [1] | YES = [1] |
|  | sp_neck; [NA] AND sp_midback; [NA]  AND  sp_lowback; [NA] | sp_neck; [NA] AND sp_midback; [NA]  AND  sp_lowback; [NA] | sp_neck; [NA] AND  sp_back; [NA] AND  no mentions of spine in the Other region open field | sp_neck; [NA] AND sp_midback; [NA]  AND  sp_lowback; [NA] | sp_neck; [NA] AND  sp_lowback; [NA] | sp_neck; [NA] AND  sp_lowback; [NA] | Missing/ not answered =  Set as NA |
| AND: for an individual to be categorised as not having experienced any spinal pain, they must have reported ‘NO’ in all three: sp_neck, sp_midback, and sp_lowback.  OR: for an individual to be categorised as having experienced any spinal pain, they must have reported ‘YES’ in any of the three: sp_neck, sp_midback, or sp_lowback. | | | | | | | |

| **sp_conseq_careseek: Have you exhibited care seeking behaviour due to spinal pain?** | | | | | | | |
| --- | --- | --- | --- | --- | --- | --- | --- |
| Cohort | DNBC 11y | DNBC 18y | G21 7y, 10y | NINFEA 13y | NFBC1986 16y | NFBC1986 18y | Values of sp_conseq_careseek |
| Item | E094; [4] | G134_3; [0] | A9; Did not select  ‘Went to a doctor’ | N9; [0] | 28-neck; [0-1] AND  28-low back; [0-1] ***** | 34-neck; [0-1] AND  34-low back; [0-1]  AND  45; if b-f are all [0]  AND  67; if b-f are all [0] ***** | NO = [0] |
|  | E094; [1-3] | G134_3; [1-3] | A9; Selected ‘Went to a doctor’ | N9; [1-3] | 28-neck; [2] OR  28-low back; [2] | 34-neck; [2] OR  34-low back; [2]  OR  45; if either b, c, d, e, f is [1] OR  67; if either b, c, d, e, f is [1] | YES = [1] |
|  | E094; not answered | G134_3; [4] OR not answered | A9; not answered | N9; not answered | 28-neck; not answered  AND  28-low back;  not answered | 34-neck; not answered  AND  34-low back; not answered AND  45; not answered  AND  67; not answered | Missing/ not answered =  Set as NA |
| * In cases where multiple values are needed in an ‘AND’ condition and some values are NA (but not all), then apply the non-NA values to determine value of sp_conseq_careseek. E.g. a child from NFBC1986 18y has the following values: 34-neck = [0], 34-low back = [1], 45 = [NA], 67 = [NA], then sp_conseq_careseek = [1] | | | | | | | |

| **sp_conseq_physact: Have you experienced physical activity restriction due to spinal pain?** | | | | | | |
| --- | --- | --- | --- | --- | --- | --- |
| Cohort | DNBC 11y | DNBC 18y | G21 7y, 10y, 13y | NINFEA 13y | NFBC1986 18y | Values of sp_conseq_physact |
| Item | E093; [4] | G134_2; [0] | A9; Did not select ‘Was unable to do their free time activities’ | N8; [0] | 47; [0]  AND  46-j; [0]  AND  68-j; [0] | NO = [0] |
|  | E093; [1-3] | G134_2; [1-3] | A9; Selected ‘Was unable to do their free time activities’ | N8; [1-3] | 47; [1-2]  OR  46-j; [1]  OR  68-j; [1] | YES = [1] |
|  | E093; not answered | G134_2; [4] OR not answered | A9; not answered | N8; not answered | 47; not answered  AND  46-j; not answered  AND  68-j; not answered | Missing/ not answered =  Set as NA |

| **sp_conseq_schoolabs: Have you experienced school absenteeism due to spinal pain?** | | | | | |
| --- | --- | --- | --- | --- | --- |
| Cohort | DNBC 11y | DNBC 18y | G21 7y, 10y, 13y | NINFEA 13y | Values of sp_conseq_schoolabs |
| Item | E092; [4] | G134_1; [0] | A9; Did not select ‘Missed classes’ | N7; [0] | NO = [0] |
|  | E092; [1-3] | G134_1; [1-3] | A9; Selected ‘Missed classes’ | N7; [1-3] | YES = [1] |
|  | E092; not answered | G134_1; [4] OR not answered | A9; not answered | N7; not answered | Missing/ not answered =  Set as NA |

| **sp_conseq: Have you had any spinal pain consequences (i.e. functional limitations)?** | | | | | | | |
| --- | --- | --- | --- | --- | --- | --- | --- |
| Cohort | DNBC 11y | DNBC 18y | G21 7y, 10y, 13y | NINFEA 13y | NFBC1986 16y | NFBC1986 18y | Values of sp_conseq |
| Item | sp_conseq_careseek; [0] AND  sp_conseq_physact;  [0] AND  sp_conseq_schoolabs; [0] | sp_conseq_careseek; [0] AND  sp_conseq_physact; [0] AND  sp_conseq_schoolabs; [0] | sp_conseq_careseek; [0] AND  sp_conseq_physact;  [0] AND  sp_conseq_schoolabs; [0] | sp_conseq_careseek; [0] AND  sp_conseq_physact; [0] AND  sp_conseq_schoolabs; [0] | sp_conseq_careseek; [0] | sp_conseq_careseek; [0] AND  sp_conseq_physact; [0] | NO = [0] |
|  | sp_conseq_careseek; [1] OR sp_conseq_physact;  [1] OR  sp_conseq_schoolabs; [1] | sp_conseq_careseek; [1] OR  sp_conseq_physact; [1] OR  sp_conseq_schoolabs; [1] | sp_conseq_careseek; [1] OR  sp_conseq_physact;  [1] OR  sp_conseq_schoolabs; [1] | sp_conseq_careseek; [1] OR  sp_conseq_physact; [1] OR  sp_conseq_schoolabs; [1] | sp_conseq_careseek; [1] | sp_conseq_careseek; [1] OR  sp_conseq_physact; [1] | YES = [1] |
|  | sp_conseq_careseek; [NA] AND  sp_conseq_physact; [NA] AND  sp_conseq_schoolabs; [NA] | sp_conseq_careseek; [NA] AND  sp_conseq_physact; [NA] AND  sp_conseq_schoolabs; [NA] | sp_conseq_careseek; [NA] AND  sp_conseq_physact; [NA] AND  sp_conseq_schoolabs; [NA] | sp_conseq_careseek; [NA] AND  sp_conseq_physact; [NA] AND  sp_conseq_schoolabs; [NA] | sp_conseq_careseek; [NA] | sp_conseq_careseek; [NA] AND  sp_conseq_physact; [NA] | Missing/ not answered =  Set as NA |
| AND: for an individual to be categorised as not having experienced any consequences of spinal pain, they must have reported ‘NO’ in all three: sp_conseq_careseek, sp_conseq_physact and sp_conseq_schoolabs. OR: for an individual to be categorised as having experienced any consequences of spinal pain, they must have reported ‘YES’ in any of the three: sp_conseq_careseek, sp_conseq_physact or sp_conseq_schoolabs | | | | | | | |

| **sp_frequency: spinal pain frequency** | | | | | | |
| --- | --- | --- | --- | --- | --- | --- |
| Cohort | DNBC 11y | DNBC 18y | G21 7y, 10y, 13y | NINFEA 13y | NFBC1986 18y | Values of sp_frequency |
| Item | E089; [3] AND  E090; [3] AND  E091; [3] * | G128; [1] AND  G130; [1] AND  G132; [1] * | A6; [0] | N1; [1]  AND  N3; [1]  AND  N5; [1] * | 42; [1]  AND  64; [1] * | Low frequency = [0] |
|  | E089; [1-2] OR  E090; [1-2] OR  E091; [1-2] | G128; [2-3] OR  G130; [2-3] OR  G132; [2-3] | A6; [1-4] | N1; [2-3]  OR  N3; [2-3]  OR  N5; [2-3] | 42; [2-4]  OR  64; [2-4] | High frequency = [1] |
|  | E089; not answered AND  E090; not answered AND  E091; not answered | G128; [4] OR not answered AND  G130; [4] OR not answered AND  G132; [4] OR not answered | A6; not answered | N1; not answered AND  N3; not answered AND  N5; not answered | 61; not answered  AND  36; not answered  AND  42; not answered  AND  64; not answered | Missing/ not answered =  Set as NA |
|  | E089; [4] AND  E090; [4] AND  E091; [4] | G128; [0] AND  G130; [0] AND  G132; [0] | A3; [did not select either neck, or back pain] | N1; [0]  AND  N3; [0]  AND  N5; [0] | 42; [0]  AND  64; [0]  AND  61; [0]  AND  36; [0] | No pain =  set as NA |
| Note: if frequency is available for several sites, choose the highest frequency reported.  * In cases where multiple values are needed in an ‘AND’ condition and some values are NA (but not all), then apply the non-NA values to determine value of sp_frequency. E.g. a child in NFBC1986, where the highest value available in 42 and 64 is [1], then sp_frequency = [0]. If the highest value available in 42 and 64 is [2] or [3], then sp_frequency = [1]. If 42 is NA, and 64 is [1], then sp_frequency = [0]. | | | | | | |

| **sp_intensity: spinal pain intensity** | | | | | | |
| --- | --- | --- | --- | --- | --- | --- |
| Cohort | DNBC 11y | DNBC 18y | G21 7y, 10y, 13y | NINFEA 13y | NFBC1986 18y | Values of sp_intensity |
| Item | E089A; [1-2] AND  E090A; [1-2] AND  E091A; [1-2] * | G129; [0-3] AND  G131; [0-3] AND  G133; [0-3] * | A7; [0-3] | N2; [1-2] AND  N4; [1-2] AND  N6; [1-2] * | 43; [0-3]  AND  65; [0-3] * | Low intensity = [0] |
|  | E089A; [3-6] OR  E090A; [3-6] OR  E091A; [3-6] | G129; [4-10] OR  G131; [4-10] OR  G133; [4-10] | A7; [4-10] | N2; [3-6]  OR  N4; [3-6]  OR  N6; [3-6] | 43; [4-10]  OR  65; [4-10] | High intensity = [1] |
|  | E089A; not answered AND  E090A; not answered AND  E091A; not answered | G129; not answered AND  G131; not answered AND  G133; not answered | A7; not answered | N2; not answered AND  N4; not answered AND N6; not answered | 43; not answered  AND  65; not answered | Missing/ not answered =  Set as NA |
| Note: if intensity is available for several sites, choose the highest intensity reported.  * In cases where multiple values are needed in an ‘AND’ condition and some values are NA (but not all), then apply the non-NA values to determine value of sp_intensity. E.g.: In NINFEA, if the highest value available in N2, N4 and N6 is [2], then sp_intensity = [0]. If the highest value available in N2, N4 and N6 is [3], then sp_intensity = [1]. If a person has N2 = [NA], N4 = [1] and N6 = [2], then sp_intensity = [0], because the highest value available is between 1-2. | | | | | | |

**Supplementary D: Harmonisation of maternal education at birth**

Maternal education at birth was harmonised across cohorts using the International Standard Classification of Education 1997 (ISCED‑97). Three categories were created to ensure comparability across national education systems: Low (ISCED 0-2, corresponding to no education to lower secondary), Medium (ISCED 3-4, corresponding to upper to post‑secondary), and High (ISCED 5-6, corresponding to bachelor’s degree and above). Because the distribution of maternal education varies across European populations and across birth cohorts, a scaled measure was constructed to capture the socioeconomic gradient in a way that is comparable across cohorts.

The scaled variable was derived from the cumulative distribution of mothers across the three education categories within each cohort. Each category was assigned a value between 0 and 1 representing the midpoint of its cumulative proportion. Specifically, mothers in the Low education group were assigned half of the proportion of Low-educated mothers in the cohort. Mothers in the Medium group were assigned the proportion of Low-educated mothers plus half of the proportion of Medium-educated mothers. Mothers in the High group were assigned the proportion of Low-educated mothers, plus the proportion of Medium-educated mothers, plus half of the proportion of High-educated mothers. For example, if 20% of mothers in a cohort had Low education, 30% Medium, and 50% High, the scaled scores would be 0.10 (0.20/2), 0.35 (0.20+0.30/2), and 0.75 (0.20+0.30+0.50/2), respectively. These values reflect the relative position of each education group within the cohort‑specific distribution.

The scaled measure was used to represent the relative socioeconomic position of each education category within the cohort-specific distribution. This approach accounts for differences in the size of the education categories across cohorts and allows socioeconomic inequalities to be compared across populations with different distributions of educational attainment. Cohort-specific distributions of maternal education (Low, Medium, High) were used to generate the scaled variable for each cohort.

**Supplementary E: Flow chart and missing values**

Figure S1: Flow chart showing how the sample size (and the percentage relative to the initial sample size) is derived at each stage in each cohort, based on the availability of measurements for any spinal pain, spinal pain consequences among children with any spinal pain, and maternal education. For the analyses, prevalence estimates were based on complete observations for the relevant spinal pain variable (shown in black in the flow chart), whereas SII and RII estimates were based on complete observations for the relevant spinal pain variable and maternal education (shown in red). The NINFEA 13-year follow-up is still ongoing. These numbers are based on data version 2024.01 (January 2024). At that time, only a subset of the cohort (children with at least 13 years of age, n~3100) was eligible to participate in the 13-year follow-up.

Table S1: Number and percentage of missing values for each variable, calculated separately for each cohort and follow-up wave.

|  | **DNBC** | | **G21** | | | **NINFEA ^c^** | **NFBC1986** | |
| --- | --- | --- | --- | --- | --- | --- | --- | --- |
| Sample size ^a^ | 96825 | | 8647 | | | 7642 | 9221 | |
| Maternal education | 31688 (32.7%) | | 56 (0.6%) | | | 66 (0.9%) | 1238 (13.4%) | |
| Follow-up | 11y | 18y | 7y | 10y | 13y | 13y | 16y | 18y |
| Any spinal pain | 50147 (51.8%) | 52145 (53.9%) | 2834 (32.8%) | 3295 (38.1%) | 4083 (47.2%) | 6141 (80.4%) | 2517 (27.3%) | 7344 (79.6%) |
| Neck pain | 50132 (51.8%) | 52467 (54.2%) | 2834 (32.8%) | 3295 (38.1%) | 4083 (47.2%) | 6146 (80.4%) | 2554 (27.7%) | 7359 (79.8%) |
| Mid-back pain | 50153 (51.8%) | 52885 (54.6%) | n/a | n/a | n/a | 6145 (80.4%) | n/a | n/a |
| Low-back pain | 50154 (51.8%) | 52721 (54.4%) | n/a | n/a | n/a | 6148 (80.5%) | 2561 (27.8%) | 7364 (79.9%) |
| Back pain ^b^ | n/a | n/a | 2834 (32.8%) | 3295 (38.1%) | 4083 (47.2%) | n/a | n/a | n/a |
| Frequency | 65040 (67.2%) | 57548 (59.4%) | 8587 (99.3%) | 8522 (98.6%) | 8347 (96.5%) | 6485 (84.9%) | 9210 (99.9%) | 7810 (84.7%) |
| Intensity | 65122 (67.3%) | 57548 (59.4%) | 8615 (99.6%) | 8582 (99.2%) | 8439 (97.6%) | 6487 (84.9%) | 9210 (99.9%) | 7737 (83.9%) |
| Any functional limitations | 50061 (51.7%) | 57822 (59.7%) | 8612  (99.6%) | 8582 (99.2%) | 8436 (97.6%) | 6140  (80.3%) | 2517 (27.3%) | 7344 (79.6%) |
| Care seeking behaviour | 50064 (51.7%) | 57665 (59.6%) | 8612  (99.6%) | 8582 (99.2%) | 8647 (100%) | 6148  (80.5%) | 2517 (27.3%) | 7344 (79.6%) |
| Physical activity restriction | 50059 (51.7%) | 57981 (59.9%) | 8612  (99.6%) | 8582 (99.2%) | 8436 (97.6%) | 6147  (80.4%) | 9210 (99.9%) | 7745 (84.0%) |
| School absenteeism | 50062 (51.7%) | 57690 (59.6%) | 8612  (99.6%) | 8582 (99.2%) | 8436 (97.6%) | 6144 (80.4%) | n/a | n/a |

n/a: not available

^a^ Missing values are based on the full cohort sample size because follow-up-specific sample sizes were not available in the harmonised data.

^b^ G21 has a composite ‘back pain’ variable of either mid- or low-back pain

^c^ The NINFEA 13-year follow-up is still ongoing, and the results presented are based on data from database version 2024.01 and therefore include only a subset of the cohort

**Supplementary F: Estimates and 95% confidence intervals of the Slope and Relative Indices of Inequality**

Figure S2: Graphical illustration of a socioeconomic gradient

Assume that in a cohort 20%, 30%, and 50% of mothers fall into the low, medium, and high educational levels, respectively. The scaled maternal education variable is then defined as the midpoint of each category in their cumulative distribution, yielding values of 0.1, 0.35, and 0.75. The scaled maternal education, $x$, is plotted against the observed prevalence of the outcome, $y$ at each educational level (see Figure S2). A simple linear regression is fitted to these three points to quantify socioeconomic inequality.

The Slope Index of Inequality (SII) is defined as the difference in predicted prevalence between the highest and lowest positions on the socioeconomic scale. It is calculated as:

$$SII=\hat{y}_{1}-\hat{y}_{0},$$

where $\hat{y}_{1}$ is the predicted prevalence at the highest socioeconomic position (scaled maternal education = 1) and $\hat{y}_{0}$ is the predicted prevalence at the lowest socioeconomic position (scaled maternal education = 0). The 95% confidence interval for the SII is calculated by

$$95\%CI \mathrm{of} SII=\left( \hat{y}_{1}-\hat{y}_{0} \right)\pm z\sqrt{Var\left( \hat{y}_{1} \right)+Var\left( \hat{y}_{0} \right)-2Cov\left( \hat{y}_{1},\hat{y}_{0} \right)},$$

where $z$ is the critical value from the standard normal distribution for a 95% confidence interval (i.e. $z=1.96$), and the variance and covariance terms are derived from the fitted linear regression model.

The Relative Index of Inequality (RII) is defined as the ratio of the predicted prevalences:

$$RII=\frac{\hat{y}_{1}}{\hat{y}_{0}}.$$

The 95% confidence interval for the RII is obtained using the Delta method, applying a first-order Taylor approximation:

$$95\%CI \mathrm{of} RII=\left( \frac{\hat{y}_{1}}{\hat{y}_{0}} \right)\pm z\sqrt{\left( \frac{\hat{y}_{1}}{\hat{y}_{0}} \right)^{2}\left[ \frac{Var(\hat{y}_{1})}{{\hat{y}_{1}}^{2}}+\frac{Var(\hat{y}_{0})}{{\hat{y}_{0}}^{2}}-\frac{2Cov(\hat{y}_{1},\hat{y}_{0})}{\hat{y}_{1}\hat{y}_{0}} \right]}.$$

**References**

1. Olsen J, Melbye M, Olsen SF, Sørensen TI, Aaby P, Nybo Andersen A-M, Taxbøl D, Hansen KD, Juhl M, Schow TB (2001) The Danish National Birth Cohort-its background, structure and aim. Scandinavian journal of public health 29:300–307

2. Strandberg-Larsen K, Cederkvist L, Aakjær A, Bjerregaard AA, Brix N, Feenstra B, Meder IK, Nohr EA, Olsen SF, Tøttenborg SS, Ramlau-Hansen CH, Vangsted A-M, Nybo Andersen A-M (2025) Cohort Profile: the Danish National Birth Cohort from foetal life to young adulthood. International Journal of Epidemiology 54

3. Correia S, Rodrigues T, Barros H (2014) Socioeconomic variations in female fertility impairment: a study in a cohort of Portuguese mothers. BMJ open 4:e003985

4. Richiardi L, Baussano I, Vizzini L, Douwes J, Pearce N, Merletti F (2007) Feasibility of recruiting a birth cohort through the Internet: the experience of the NINFEA cohort. European journal of epidemiology 22:831–837

5. Järvelin MR, Hartikainen‐Sorri AL, Rantakallio P (1993) Labour induction policy in hospitals of different levels of specialisation. BJOG: An International Journal of Obstetrics & Gynaecology 100:310–315

6. Järvelin MR, Elliott P, Kleinschmidt I, Martuzzi M, Grundy C, Hartikainen AL, Rantakallio P (1997) Ecological and individual predictors of birthweight in a northern Finland birth cohort 1986. Paediatric and perinatal epidemiology 11:298–312

7. Auvinen J, Eskola PJ, Ohtonen H-R, Paananen M, Jokelainen J, Timonen M, Vahtera J, Leino-Arjas P, Karppinen J (2017) Long-term adolescent multi-site musculoskeletal pain is associated with psychological distress and anxiety. Journal of Psychosomatic Research 93:28–32

8. University of Oulu (2020) Northern Finland Birth Cohort 1986 (Version 1). University of Oulu, Oulu
